# Design characteristics and inclusion of evidence-based exercise recommendation in fall prevention community exercise programs for older adults in Canada: a national descriptive self-report study

**DOI:** 10.1186/s12877-020-01949-2

**Published:** 2021-01-09

**Authors:** Alexie J. Touchette, Alison R. Oates, Verena H. Menec, Kathryn M. Sibley

**Affiliations:** 1 Department of Community Health Sciences, Rady Faculty of Health Sciences, University of Manitoba, Winnipeg, MB Canada; 2 College of Kinesiology, University of Saskatchewan, Saskatoon, SK Canada; 3 George and Fay Yee Centre for Healthcare Innovation, 379 – 753 McDermot Avenue, Winnipeg, MB R3E 0T6 Canada

**Keywords:** Fall prevention, Older adults, Balance, Evidence-based recommendations, Community exercise

## Abstract

**Background:**

Training balance through exercise is an effective strategy to reduce falls in community-dwelling older adults. Evidence-based fall prevention exercise recommendations have been proposed, specifying that exercise programs should: (1) provide a high challenge to balance, (2) be offered for a least three hours per week, (3) be provided on an ongoing basis. Community exercise programs have the potential to deliver effective fall prevention exercise; however, current design characteristics and whether they include the recommendations is not known. This study described design characteristics of fall prevention community exercise programs for older adults (50 years and older) across Canada, and explored whether these programs included the three evidence-based exercise recommendations.

**Methods:**

Instructors of fall prevention community exercise programs completed electronic self-report questionnaires following a modified Dillman recruitment approach. Questions explored program characteristics, exercise content, target population, and program and instructor demographic information. Using a previously developed coding scheme based on recommendations, exercises were coded for balance challenge.

**Results:**

One hundred fourty completed eligible questionnaires were analyzed (74% response rate). One hundred thirty-three programs (95%) included the challenge recommendation by prescribing mostly moderate or high challenge balance exercises, 16 programs (11%) included at least three hours of exercise a week, and 59 programs (42%) were offered on an ongoing basis. Eight programs (6%) included all three recommendations.

**Conclusions:**

Most programs included at least one recommendation for effective fall prevention exercise. Future studies should examine organizational barriers and facilitators to incorporating evidence-based exercise recommendations and explore the use of mixed home/in-class strategies to include the recommendations.

## Background

The importance of preventing falls and associated injuries among older adults, many of whom live independently in community settings, is well-recognized [1]. Extensive evidence highlights the role of exercise, specifically exercise that trains balance, in preventing falls in community-dwelling older adults. A 2017 systematic review of 283 randomized controlled trials (159,910 participants) concluded that exercise is likely the most effective intervention to reduce falls and associated injuries [2]. Most recently, a 2019 Cochrane review of 108 fall prevention exercise studies (23,407 participants) demonstrated that balance exercise reduced the number of people falling by 13% and the rate of falls by 24% [3]. Complementary meta-analyses examining the effects of specific exercise design characteristics on fall rates have informed the development of evidence-based exercise recommendations for fall prevention [4]. These recommendations specify that exercise should 1) provide a high challenge to balance through reducing the base of support, moving the centre of mass and controlling body position while standing, and standing without arm support, 2) be conducted for at least three hours per week, and 3) be offered on an ongoing basis [4].

Community exercise programs – broadly defined as publicly or privately funded group programs that are easily accessible to people living within the community with the intention to promote the health and well-being of its members [5–9] – are a potential delivery mode for implementing evidence-based fall prevention exercise recommendations. If community exercise programs include effective fall prevention exercise, they could influence the health of community-dwelling older adults. Globally, efforts have been made to identify fall prevention exercise resources and challenges associated with their implementation. For example, a 2010 Canadian report identified 282 fall prevention initiatives, 205 of which included exercise [10]. In the United States, the Centre for Disease Control and Prevention United States published a synthesis of effective fall prevention exercise programs for community-dwelling older adults [11]. A 2019 Centre for Ageing Better report highlighted barriers to implementing community exercise programs for older adults across England, and associated strategies to overcome these [12]. Although these reports provide valuable insight, detailed information on exercise design and content of existing community exercise programs has yet to be examined, thus highlighting an important gap in the literature. Although a 2019 Winnipeg, Manitoba self-report survey study which aimed to describe older adult community exercise program design found that most programs represented in the survey did not explicitly focus on fall prevention and did not include all exercise recommendations, the scope was limited to just one city in Canada [13].

The aim of this study was to describe program design characteristics of fall prevention community exercise programs for adults aged 50 years and older in Canada, and determine whether they include evidence-based exercise recommendations for fall prevention. Understanding the current state of practice and opportunities for improvement are a critical foundational component of implementation research [14], as it can help identify strengths of existing programs, as well as gaps to address in subsequent interventions.

## Methods

### Study design

A full description of the methods is presented elsewhere in partial fulfillment of the requirements of the degree of Master of Science at the University of Manitoba [15]. This cross-sectional self-report study was conducted in 2019 through an electronic survey questionnaire approach. The Checklist for Reporting Results of Internet E-Surveys (CHERRIES) recommendations, developed to ensure complete descriptions and quality of reporting electronic survey methodology was adopted where appropriate (e.g., study design, development and administration of the questionnaire, recruitment and sample description, analysis) [16]. Ethics approval was obtained from the University of Manitoba Health Research Ethics board (HS22364).

### Participants

Instructors of Canadian group exercise programs targeting community-dwelling older adults (≥50 years and living outside of government-funded institutions), that took place within the community, and that specified fall prevention or balance training as a primary goal were eligible for this study. The minimum program age of 50 years was established to include programs with a broad concept of “older adults”. Potential participants were identified directly from a multi-phase online search of “fall prevention” and “exercise classes” that identified potentially relevant programs or indirectly through referrals from related contacts (e.g., program coordinators) identified through the online search. The first page of the questionnaire consisted of the consent disclosure form. Informed consent was assumed for participants who moved forward in the questionnaire.

### Procedure

Data collection occurred between May 2019 and July 2019. A modified Dillman recruitment approach [17] was used to contact potentially eligible participants and related contacts (e.g., program coordinators). Recruitment included four contact attempts over the course of four weeks: a prenotice e-mail, distribution of the questionnaire sent a week after the prenotice, and a follow-up reminder to non-responders every week for two consecutive weeks.

### Questionnaire instrument

The electronic questionnaire was adapted from a previous, similar study [13] and questions were informed by aspects of the fundamental principles of exercise design (i.e., frequency, intensity, time, type) [18]. The questionnaire was piloted through an iterative process in which each participant received an updated version of the questionnaire based on previous feedback with 14 participants from five Canadian provinces, identified and recruited using a snowball sampling strategy from research team contacts.

The final questionnaire [see Additional file 1] contained five sections with open- and closed-ended questions. The first section confirmed eligibility (three questions). If participants were eligible, they proceeded to the program design section (21 questions). Variables in this section explored frequency, duration, and length of classes; balance challenge; prescription of home exercises; and provision of class/home resources. The third section (six questions) explored exercise content through a list of 17 standing balance exercises, 17 walking exercises, and five strengthening exercises. Instructors were asked to indicate whether they conducted the exercises in the lists provided and to specify the form in which the exercises were completed (e.g., with or without support). The fourth section (four questions) focused on program target population and inclusion/exclusion criteria, and the fifth section (eight questions) explored program and instructor demographic information (e.g., program location, instructor title/role, education background, training in falls prevention in general, years of experience).

The questionnaire and all communications with participants were available in both official Canadian languages (English and French). In order to address the possibility of instructors teaching multiple classes/sessions within a single program or an instructor teaching multiple programs, participants were instructed to answer the questions for all their classes/sessions as a whole, rather than answering for one specific class/session. An open-ended question allowed instructors to share additional information at the end of each section.

### Data processing and analysis

Incomplete questionnaire responses (i.e., missing more than one full section or missing variables used to calculate two or more of the three recommendation variables) were not included in the analysis. Open-ended questions and “other” response options were reviewed and coded into categories when appropriate. If responses were unclear or there were fewer than five observations per case, responses were kept as “other” or combined if appropriate. Responses from the provinces of Nova Scotia and New Brunswick were combined due to a small (*n* = 5) number of responses. Questionnaire variables were used directly or indirectly through the creation of derived variables to determine whether the programs included the three exercise recommendations (e.g., frequency and length of class to calculate total exercise time per week). Urban and rural setting were manually assigned by a web-based list of Canadian postal codes [19] and their respective city/municipal area. Total balance exercise time was calculated from the frequency of classes and coded open-ended responses. Most frequently prescribed exercises were those that were prescribed by at least three quarters (n= > 105) of programs (regardless of form). Exercises were assigned a balance challenge score (low/ moderate/ high), based on an existing coding scheme [13].

Inclusion of each exercise recommendation was coded into dichotomous variables (i.e., yes/no) and defined as: 1) conducting a total of at least three hours of exercise per week; 2) prescribing mostly (> = 50%) moderate to high balance challenge exercises; 3) and being offered on an ongoing basis (i.e., offered continually throughout the year) with no restrictions on number of times an individual could register for the program. Data were summarized using descriptive statistics (frequencies, proportions, ranges) in Excel.

## Results

### Recruitment (Fig. 1 and Table 1)

A total of 453 potentially eligible programs were identified through the multi-phase online search. Given that many programs listed one contact person for multiple programs and locations, this equated to 334 persons to contact (24 of whom were instructors who were contacted directly, and 310 of whom were other related contacts, such as program coordinators). The latter either forwarded the study’s information to their instructors, who then contacted the PI if they were interested in participating, or provided their instructors’ contact information to the PI to be contacted. This recruitment approach led to the identification of 232 instructors who were sent the survey link. Over 73% (*n* = 171) of participants returned the survey, 21 of which were not eligible based on participant responses, and 10 were incomplete. Analysis was conducted on 140 questionnaires.

**Fig. 1:**
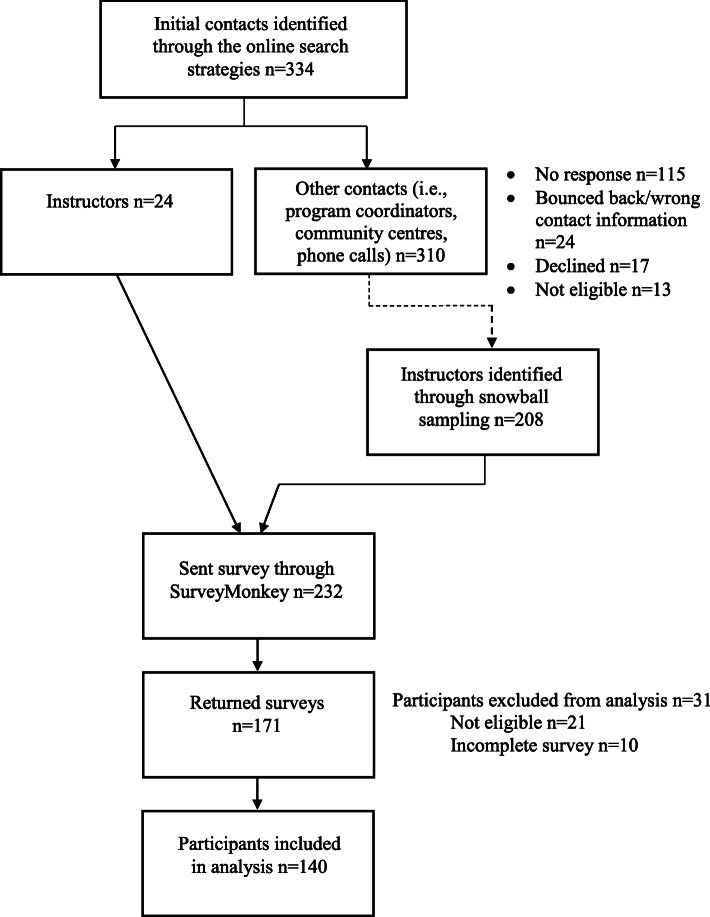
Recruitment flowchart. No response refers to those contact persons who had not responded at all and those who had initially responded with a question or comment but did not respond after the PI had replied to their message and had attempted to follow-up after a week. The dotted line indicates the non-linear process in which instructors were identified through program coordinators, other instructors, and community centres forwarding the study’s information, or the PI receiving the instructor’s e-mail addresses from the other contacts

**Table 1:** Demographic Information for Program Location and Setting

Province	Number of programs	Percent of programs
British Columbia	20	14%
Alberta	14	10%
Saskatchewan	15	11%
Manitoba	13	9%
Ontario	66	47%
Quebec	6	4%
Nova Scotia and New Brunswick*	5	4%
Missing	1	0.7%
Setting		
Urban	92	66%
Rural	34	24%
Both	8	6%
Missing/Unclear	6	4%

*Note. No responses were received from two provinces (Newfoundland and Labrador; Prince Edward Island) or the three territories (Nunavut; Yukon, and Northwest Territories)

### Instructor and program characteristics (Table 2)

Participants reported a range of educational backgrounds, and the majority reported receiving training in falls prevention in general (*n* = 109, 78%). Most reported having four or more years of experience in their role (*n* = 73, 52%), and that the program was delivered by a certified fitness professional (*n* = 69, 49%) or a health professional (*n* = 34, 24%).

**Table 2:** Summary of Instructor and Program Characteristics

Characteristics	Number of programs	Percent of programs
Instructor educational background^*a*^		
Certification	81	58%
Health care professional	62	44%
Other fitness/sports/physical training	22	16%
Specific fall prevention/older adult training	16	11%
Education/teaching degree	12	9%
None	8	6%
Other	6	4%
Specific training or education in falls prevention		
Yes	109	78%
No	30	21%
Missing	1	0.7%
Years of experience in their role		
1 year or less	25	18%
2 years	17	12%
3 years	24	17%
4 years	17	12%
5 years	13	9%
6 years or more	43	31%
Missing	1	0.7%
Program delivered by		
Certified fitness professional	69	49%
Health professional	34	24%
Peer-leader	29	21%
Other	7	5%
Missing	1	0.7%
Specific older adult population targeted		
No	21	15%
Yes	119	85%
Healthy older adults*	48	40%
Older adults with a previous fall history*	41	34%
Older adults with a specific health condition*	20	17%
All of the above*	47	39%
Others:*	16	13%
Anyone at risk of falls/trouble with balance*	9	8%
Anyone with mobility difficulties*	7	6%
Specific inclusion criteria		
No	78	56%
Yes	62	44%
Minimum strength level**	61	98%
Completion of medical clearance**	35	56%
Minimum performance of specific tasks**	48	77%
Minimum independence level**	27	44%
No serious/unstable medical or neurological disorders**	5	8%
Other (age, transportation, language, income, falls history/risk)**	7	11%
Exercise frequency (# classes/week)		
1 per week	33	24%
2 per week	83	59%
3 per week	10	7%
4 or more per week	8	6%
Other/unclear	6	4%
Class length (in hours)		
< 1 h	39	28%
1 h	81	58%
> 1 h - < 2 h	17	12%
2 h	2	1%
Other/unclear	1	0.7%
Total exercise time (in hours) per week		
< 1 h	7	5%
1 h	20	14%
> 1 h - < 2 h	23	16%
2 h	56	40%
> 2 h - < 3 h	12	9%
3 h or more	16	11%
Unclear	6	4%
Prescription of home exercises		
Yes	93	66%
No	47	35%
Provision of class/home resources		
Yes	93	66%
No	47	35%
Program duration		
Continually throughout the year	63	45%
Fixed period of time	77	55%
Progression of balance exercise challenge over the duration of the exercise program		
Stays the same	27	19%
Becomes less challenging	4	3%
Becomes more challenging	109	78%
Provision of options to allow participants to make the exercises more or less challenging		
Yes	135	96%
No and Other	5	4%

^*a*^*Note.* The educational background question in the survey was a “check all that apply” format, accounting for the discrepancies between instructor education background and program delivery

*Note. Proportions calculated based on total number of programs that targeted a specific older adult population (*n* = 119)

**Note. Proportions calculated based on total number of programs that had specific inclusion/exclusion criteria (*n* = 62)

Most participants (*n* = 119, 85%) reported that their program targeted at least one specific older adult population (e.g., healthy older adults, older adults with a previous fall history), but more than half (*n* = 78, 56%) did not specify inclusion/exclusion criteria. Based on participant responses, nearly 60% of programs offered classes twice a week (*n* = 83, 59%), and for one hour per class (*n* = 81, 58%). Two-thirds of participants (*n* = 93, 66%) reported prescribing home exercises and providing class/home resources. Of the programs that were offered continually throughout the year (*n* = 63, 45%), four programs (6%) restricted the number of times an individual could sign up for the program. Regarding balance exercise challenge, most participants (*n* = 109, 78%) reported that exercises became more challenging over time and that options were provided to make exercises more or less challenging (*n* = 135, 96%). Based on participant responses, 21 exercises (54%) were conducted in at least three-quarters of programs (Table 3).

**Table 3:** Most Frequently Prescribed Exercises and Their Challenge Scores

Exercises and Form	Number of Programs (%)		Challenge Score (max = 5)	
	The majority perform with arm support	The majority perform without arm support	With arm support	Without arm support
Sit to stand (up from chair)	31(22%)	107 (76%)	3	4
Raising arms- any direction	20 (14%)	115 (82%)	1	2
Heel raises	70 (50%)	63 (45%)	2	4
One-legged stance	91 (65%)	42 (30%)	1	3
Basic standing, focused on not leaning/staying upright relative to the floor/gravity	23 (16%)	108 (77%)	0	1
Walking (comfortable pace)	10 (7%)	121 (86%)	2	3
Basic standing comfortable position	14 (10%)	116 (83%)	0	1
Standing narrow stance	40 (29%)	89 (64%)	1	2
Standing tandem (toe-heel directly in front of one another)	64 (46%)	65 (46%)	1	3
Standing wide stance	13 (9%)	115 (82%)	0	1
Shifting weight as far as possible in either direction	60 (43%)	68 (49%)	1	3
Hip strategy weight shifts	52 (37%)	70 (50%)	1	3
Ankle strategy weight shifts	55 (39%)	67 (48%)	1	3
Walking sideways- side steps	18 (13%)	98 (70%)	2	3
Heel to toe (tandem) walking	45 (32%)	67 (48%)	3	4
Walking while talking	12 (9%)	99 (71%)	1	3
Walking and changing directions (i.e., a turn of more than 45 degrees)	22 (16%)	83 (59%)	3	5
Strength Exercises and Forms	The majority perform while sitting	The majority perform while standing		
Legs (e.g., squats, lunges, etc.)	16 (11%)	117 (84%)	N/A	
Arms (e.g., bicep curl, triceps extension, etc.)	50 (36%)	77 (55%)	N/A	
Chest (e.g., wall push-ups, chest press, etc.)	31 (22%)	92 (66%)	N/A	
Shoulders (e.g., overhead press, deltoid lateral raise, etc.)	57 (41%)	65 (46%)	N/A	
Core (e.g., plank, seated ab crunch, rows, etc.)	91 (65%)	24 (17%)	N/A	

### Inclusion of evidence-based exercise recommendations (Table 4)

Based on the results from the coded exercises, almost all programs (*n* = 133, 95%) prescribed mostly (> = 50%) moderate or high challenge balance exercises. Regarding the time recommendation, just over 10% of programs (*n* = 16, 11%) reported conducting three hours or more of exercise per week. Based on a stricter interpretation of the recommendation (i.e., total balance exercise time per week), a wide range of total balance exercise time per week was reported (5–200 min), with only one program (0.7%) including three hours per week focused on balance. Based on responses, less than half of programs included the recommendation of being offered on an ongoing basis with no restrictions on individuals’ registration (*n* = 59, 42%). Among the 140 completed questionnaires, more than half of programs (*n* = 74, 53%) included only one of the three recommendations, 55 programs (39%) included only two recommendations (regardless of which two), eight programs (6%) included all three, and three programs (2%) included none.

**Table 4:** Summary of Programs Including Effective Fall Prevention Exercise Recommendations

Recommendations	Number of programs	Percent of programs
Moderate to high challenge to balance	133	95%
At least 3 h of exercise per week	16	11%
Offered on an ongoing basis	59	42%

*Note.* Eight programs (6%) included all three recommendations

## Discussion

To our knowledge, this is the first national investigation of existing fall prevention community exercise programs for older adults. An important finding was the varied distribution of evidence-based exercise recommendations in the represented programs– virtually all programs (95%) included moderate or high challenge to balance, just over 10% of programs (11%) included the recommended three hours of exercise per week, and just less than half of programs (42%) were offered on an ongoing basis.

The challenge recommendation was met by almost all the programs represented in this study. This is important because more challenging exercise programs have larger preventative effects on falls [4]. However, while the coding framework used included either moderate or high challenge exercises as meeting the balance challenge recommendations, we note that most of the prescribed exercises were a moderate challenge to balance. Balance challenge is not yet well operationalized in practice as compared to cardiovascular or strength training, which have established methods (e.g., instruments and scales) for documenting challenge and progression. In their recent work, Farlie and colleagues [20] have developed and conducted the initial validation of two new balance challenge measures – the Balance Intensity Scale for Therapists (BIS-T) and the Balance Intensity Scale for Exercisers (BIS-E). These measures were developed through systematic observational analysis of performance of balance tasks and through stakeholder consultation. Although these efforts to operationalize and systematically measure balance challenge are ongoing in the literature, instructors play a critical role in ensuring that the challenge level is sufficient for a training effect. It is therefore recommended that instructors evaluate challenge level through active monitoring in classes (e.g., giving cues, asking participants to rate the difficulty level of exercises), assessment using the BIS-E, and tracking falls over time to explore program effectiveness.

Just over 10% of programs represented in this study included the recommended three hours of exercise per week, with just one program including three hours of balance specific exercise per week. This indicates that currently, in Canada, community exercise classes alone are unlikely to include the recommended dose of exercise needed to prevent falls. This study did not examine program planning and design considerations, therefore it is not possible to speculate why a low number of programs included this recommendation, however, challenges to implementing fall prevention programs have been reported in the literature [21]. The finding that almost a quarter of instructors reported not having received specific training in falls prevention and that just over 20% of instructors were peer-leaders may also be acting as a barrier to implementation as the role of education has been found to be important in the implementation of evidence-based practice in physical therapy [22, 23]. Although a recent systematic review and meta-analysis of the effectiveness of peer-led exercise programs for community-dwelling older adults reported inconclusive results regarding improved physical outcomes due to small sample sizes (3492 participants), the authors found that peer leaders can help promote and maintain adherence to exercise programs [24]. Another report highlights that including trained volunteers can be beneficial for the volunteers as well as for the clients [12]. Further investigation into the influence of instructor education and training on implementation of evidence-based practice is needed: Training peer-leaders to deliver programs could have important implications for reaching more older adults by increasing the number of available programs, especially in remote or rural areas. Other commonly cited organizational barriers to providing evidence-based practice in the literature include a lack of available and appropriate community venues [12], the number of full time staff, funding [22], and unavailable tools and lack of equipment [23]. Therefore, including the time recommendation may not be feasible for some programs. Future research exploring personal, organizational, and systemic barriers to including three hours of total and balance-specific exercise per week is warranted, as it is critical for instructors to receive the necessary organizational support to deliver evidence-based programs [12]. Furthermore, given that most programs prescribed home exercises and home/class resources, future studies should explore these exercise characteristics and practices. Prescribed home exercise programs should provide a high challenge to balance, offer additional home exercise resources (e.g., home visits, videos of exercises, exercise journal, etc.), and be monitored and tracked in order to encourage and support participants in completing the recommended exercise dose beyond the group exercise class. Although this additional support for home exercises may involve increased financial resources, thus acting as another significant barrier to implementation, further exploration into this possibility is warranted in order to reach the recommended dose for effective fall prevention exercises.

A little less than half of included programs were offered on an ongoing basis, thus meeting the duration recommendation. This provides important insight into program design as ongoing exercise is crucial for sustained effects [4]. Future work should focus on program decision-making and collaborating with program coordinators to explore potential options for sustained delivery. Given that the findings from the current study suggest that individual classes are unlikely to include all exercise recommendations to prevent falls, we suggest that multiple strategies, including a combination of in-person and home-based exercises are needed. For example, an audit of 35 exercise groups randomly selected from 714 eligible exercise classes in Australia found that, while considered alone, no in-person program met their recommended criteria, but when the home exercise component was also considered, 23% of programs met their criteria [25]. A potential option may be for programs to include one in-person class per week and a carefully monitored home program that provides appropriate support for individuals to exercise safely and effectively at home. This may allow programs to operate more continuously. Established effective home based programs, such as the individually tailored physiotherapist or trained instructor led Otago program [26] and the FallProof™ At Home Exercise Program DVD [27], may be used by community programs as a guide. However, it is important to note that progression in the level of challenge of exercises is a key component to promoting improvements and benefits of an exercise program [12]. As such, home based programs (digital or not) should be developed to progress over time and for different levels of ability in order to appropriately challenge exercisers.

### Limitations and considerations

The self-report nature of the survey methodology was the primary limitation of this study [28, 29]. Additionally, although efforts were made to identify as many potentially eligible fall prevention community programs as possible through the online searches and snowball sampling, it is important to note that the total number of instructors or programs in Canada is not known, as there may be programs that do not have an online presence or are offered only through referrals. Furthermore, we were unable to recruit an instructor for all programs identified through the online search, as some instructors and other related contacts declined to participate or did not respond.

Furthermore, although most provinces were represented in this study, no responses were received from the three territories (Northwest Territories, Nunavut, and Yukon) and two provinces (Newfoundland and Labrador, and Prince Edward Island). Given the different provincial contexts for administering community exercise programs (e.g., provincial funding, privatized recreation centres), the survey had a general structure, meaning that some questions may not have been relevant to each participant within their specific provincial context. Long-term observation and qualitative interviews could offer more insight into different provincial contexts. Furthermore, this study was conducted in Canada and can only be interpreted within the Canadian context. Therefore, it is recommended that other countries replicate the survey in order to explore the state of their fall prevention community exercise programs.

## Conclusion

This study provides insight into design characteristics of fall prevention community exercise programs in Canada. Exercise programs should include at least three hours per week of high challenge exercise, on an ongoing basis [4]. Although most programs in the current study did not include all three recommendations, most included a moderate to high challenge to balance. Identifying these existing strengths, as well as any gaps is an important step to support implementation of effective fall prevention exercise. Therefore, we recommend that future research should investigate barriers and facilitators to the inclusion of evidence-based recommendations in the design of fall prevention community exercise programs at the organizational level, in addition to developing and measuring augmented program formats.

## Supplementary Information

**Additional file 1.** Questionnaire Instrument. The questionnaire instrument developed and used in this study.

## Data Availability

The datasets used and analyzed during the current study are available from the corresponding author on reasonable request.

## Abbreviations

CHERRIES: Checklist for Reporting Results of Internet E-Surveys
BIS-T: Balance Intensity Scale for Therapists
BIS-E: Balance Intensity Scale for Exercisers
