# Supplementary material for: Design characteristics and inclusion of evidence-based exercise recommendation in fall prevention community exercise programs for older adults in Canada: a national descriptive self-report study
Source: BMC Geriatr. 2021 Jan 9;21:33. doi: 10.1186/s12877-020-01949-2 (PMC7796610; doi:10.1186/s12877-020-01949-2)
Supplement: Supplementary file 1 — Additional file 1. Questionnaire Instrument. The questionnaire instrument developed and used in this study. [file 12877_2020_1949_MOESM1_ESM.docx]

Additional File 1: Questionnaire Instrument

Consent Disclosure

**Thank-you for accessing the Understanding Current Fall Prevention Program Design in Community- Based Exercise Programs for Older Adults in Canada online survey. This survey is part of a**

**research study conducted at the University of Manitoba as partial fulfillment of the Masters of**

**Science program with Community Health Sciences.**

**This survey is being conducted to describe characteristics of fall prevention and balance training exercise programs for community-dwelling older adults living independently outside of government-funded healthcare (aged 50 years and older) in Canada.**

**Information about the exercise program you are affiliated with will be collected through an online survey which will ask you a series of questions and should take approximately between 15-25 minutes to complete.**

**Your participation in this survey is completely voluntary. You are not required to provide any personal information such as your name, address or telephone number, and you don’t have to answer any questions you don’t want to. All respondent information (i.e., name, e-mail addresses, IP addresses, and program name) will not be collected into the survey results.**

**The risks of participating are low. Possible risks include tiring from answering questions and potential loss of confidentiality, though precautions are in place to reduce these risks.**

**If you agree to participate in the survey, the survey system will automatically save your progress so you can close the survey and return to complete it at a later time. If you choose to do this, to get back to the survey click on the “Begin Survey” link in the recruitment e-mail that was sent to you.**

**Please note that when you submit your response, you will not be able to withdraw or change them as we cannot link the survey responses back to you.**

**Information from this study may be published and/or presented in public forums, but your name and the name of your associated exercise program will not be used or disclosed. All participating programs and participants will be assigned a unique study ID. No names or identifying information will be collected. All information provided will be kept confidential and will only be used for research purposes. All information will be kept for 5 years after completion of the study in case further analysis is needed. After 5 years, physical information will be destroyed via shredding and digital information will be deleted from hard drives.**

**Your participation is important to us and will give us valuable insight on the resources available for community dwelling older adults interested in fall prevention and balance training exercise programs. Information from this study will be used to understand current practices in older adult**

**fall prevention and balance training community exercise programs. If you have any questions about this survey study, please do not hesitate to contact _________, at __________________ or at __________________ or _________ at __________________ or _________________.**

Consent Disclosure

**This study is funded in part by the Canada Research Chairs program through ________ Canada**

**Research Chair in Integrated Knowledge Translation in Rehabilitation Sciences.**

**This study has been approved by the University of Manitoba Health Research Ethics Board.**

**By continuing on and completing the online survey you are consenting to participate in the online survey.**

Section 1: Eligibility

**Thank you for agreeing to participate in this study. The purpose of the study is to describe characteristics of fall prevention and balance training exercise programs for community-dwelling older adults living independently outside of government-funded healthcare (aged 50 years and older) in Canada. Your participation is highly valued. Please answer the following questions as truthfully as possible.**

**If you teach multiple sessions/classes of the exercise program identified in the recruitment e-mail and throughout the survey, please think of all the sessions you teach of that program as a whole (i.e., not individual sessions/classes) when answering the questions.**

1. Is fall prevention and/or improving balance a primary goal of the {{ contact.custom1 }} exercise program?


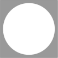
 Yes


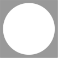
 No

2. Is the {{ contact.custom1 }} exercise program directed for community-dwelling older adults (any age group of at least 50 years or older)?


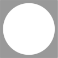
 Yes


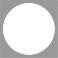
 No

3. Are you a primary instructor of the {{ contact.custom1 }} exercise program? The primary instructor is the individual who may be in charge of planning, coordinating and developing class content, teaching the majority of classes while monitoring individual progress and offering support and assistance, etc.


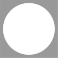
 Yes


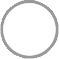
No

Section 2: Program Design

**The next questions refer to the design and delivery of the exercise program. Please check the best answer and specify if needed.**

**If you teach multiple sessions/classes of the exercise program identified in the recruitment e-mail and throughout the survey, please think of all the sessions you teach of that program as a whole (i.e., not individual sessions/classes) when answering the questions.**

4. How many sessions/groups of the {{ contact.custom1 }} do you teach?


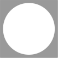
 1
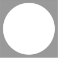
 2


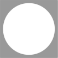
 3 or more

5. How often are classes conducted per week?


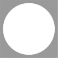
 Once per week


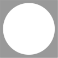
 Twice per week


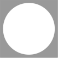
 Three times per week


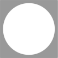
 Four times per week


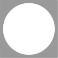
 Five or more times per week


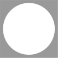
 Other, please specify:

6. How long is each class in hours?


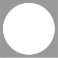
 0.5 hours
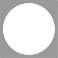
 0.75 hours
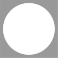
 1 hour


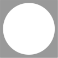
 1.25 hours
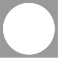
 1.5 hours
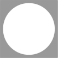
 1.75 hours
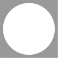
 2 hours


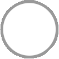
Other (please specify)

7. How long is the {{ contact.custom1 }} exercise program offered?


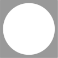
 Continually throughout the year


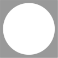
 For a fixed period of time (e.g. 12 week sessions offered 3 times a year).

8. Is there a maximum number of times that an individual can register for the {{ contact.custom1 }} exercise program?


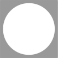
 No


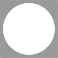
 Yes

Section 2: Program Design

9. Please specify how many times a year the {{ contact.custom1 }} exercise program is offered and for how

many weeks:

10. Please specify the maximum number of times that an individual can register for the {{ contact.custom1

}} exercise program:

11. Is there anything else you would like to tell us about the delivery of the {{ contact.custom1 }} exercise

program?

Section 2: Program Design

**The next questions ask about the portion of the exercise program focused on fall prevention and/**

**or improving balance. Please check the best answer and specify if needed.**

12. Are there significant differences in the fitness/ functional level of participants in the different sessions/ groups of the {{ contact.custom1 }} program that you teach (i.e., session/group A consists of older adults with lower functional level and session/group B consists of older adults with higher functional level)?


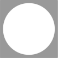
 Yes


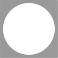
 No

13. In a typical class, how much time in minutes is spent on exercises targeting balance in standing or walking?

14. When prescribing balance exercises, are options provided to allow participants to make the exercises more or less challenging?


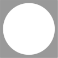
 Yes, options are provided


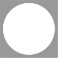
 No, everyone does the same exercise


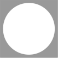
 Other, please describe:

15. In general, how does the level of balance challenge change over the duration of the {{ contact.custom1

}} exercise program?


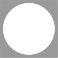
 Stays the same


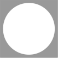
 Becomes more challenging


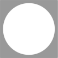
 Becomes less challenging

16. What is the primary way in which you determine how challenging the balance exercises are for the participants?


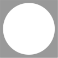
 Based on recommendation/ prescription of a doctor or physical therapist


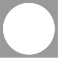
 Based on participant's successful performance of previously completed balance exercises


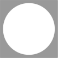
 Participant's decision


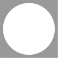
 As weeks progress, challenge increases


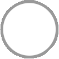
Other, please specify:

17. In your opinion, do the majority (50% or more) of participants experience exercises which:


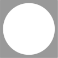
 Fully challenge balance (i.e., the balance exercises performed near the limits of postural stability)
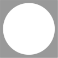
 Do not fully challenge balance or challenge balance only in a minority (<50%) of exercises


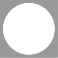
 Never challenge balance

18. During a typical balance exercise section of the {{ contact.custom1 }} exercise program, do you see any of the following behaviours in your participants? Check all that apply.

No- not seen

Yes- seen in the majority (>=50%)

Yes- seen in the minority (<50%)


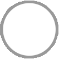

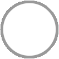

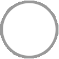


Increased sway compared with resting position


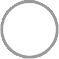

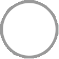

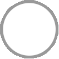
Ankle strategy (small corrective balance reaction resulting primarily from movement at the ankle- completed without taking a step)


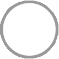

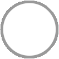

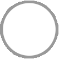


Hip strategy (small corrective balance reaction resulting primarily from movement at the hip- completed without taking a step)

Step strategy (taking a step to regain balance/prevent a fall)
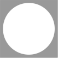

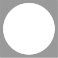

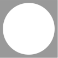
 Reaching (towards something/someone else to hold on to)


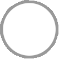

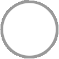

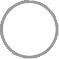


Flailing arms
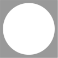

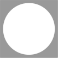

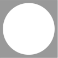


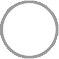

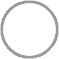

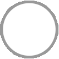


Holding arms, legs, or trunk stiff in any position

Making fist(s)
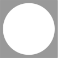

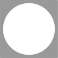

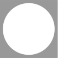
 Pulling/tugging on own clothing


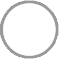

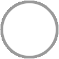

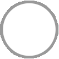


Other, please specify:

19. Is there anything else you would like to tell us about the fall prevention and/or improving balance focus

of the {{ contact.custom1 }} exercise program?

Section 2: Program Design

**The next questions ask about home exercise prescription. Please check the best answer and specify if needed.**

20. Do you prescribe home exercises to participants?


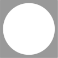
 No


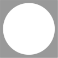
 Yes

21. Do you provide out of class/home resources to participants?


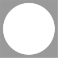
 No


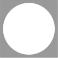
 Yes

Section 2: Program Design

22. Please specify **what home exercises** are prescribed to participants and **how often** they are

prescribed:

23. Please specify the out of class/home resources that are provided to participants:

24. Is there anything else you would like to tell us about the home exercise prescription portion of the {{

contact.custom1 }} exercise program?

Section 3: Exercise Content

**The next section will focus on the exercise content of the exercise program. For each exercise, please check the appropriate boxes and specify if needed.**

**If you teach multiple sessions/classes of the exercise program identified in the recruitment e-mail and throughout the survey, please think of all the sessions you teach of that program as a whole (i.e., not individual sessions/classes) when answering the questions.**

25. In a typical class, which of the following **standing balance exercises** do the majority (>=50%) of your participants perform?

If yes, please check whether the majority (>=50%) of participants perform the exercise with or without arm support (i.e., chair, counter, wall, cane). Please note that support may be available for safety reasons.

Yes- and the majority perform **with arm support** (i.e., chair, counter,

No wall)

Yes- and the majority

perform **without arm support** (support may be

available for safety)


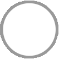

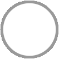

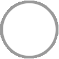


Basic standing, focused on not leaning/staying upright relative to the floor/gravity

Basic standing comfortable position
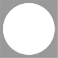

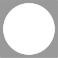

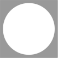


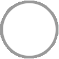

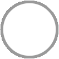

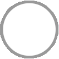


Standing wide stance

Standing narrow stance
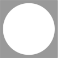

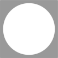

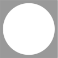


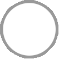

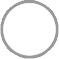

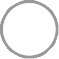


Standing tandem (toe-heel directly in front of one another)

One-legged stance
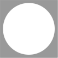

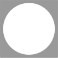

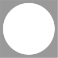


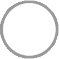

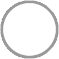

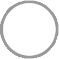


Shifting weight as far as possible in either direction

Standing with eyes closed
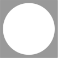

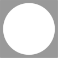

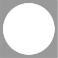


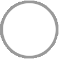

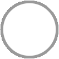


Toe taps on bench step- any direction

Sit to stand (up from chair) Raising arms- any direction

Heel raises

Hip strategy weight shifts

Ankle strategy weight shifts

Obstacle course

Pushing/nudging/perturbing/throwing off balance

Catching ball or other projectile

Other (please specify)

26. In a typical class, which of the following **walking balance exercises** do the majority (>=50%) of your participants perform?

If yes, please check whether the majority (>=50%) of participants perform the exercise with or without arm support (i.e., chair, counter, wall, cane). Please note that support may be available for safety reasons.

Yes- and the majority

perform **with arm support** (i.e., chair,

No counter, wall)

Yes- and the majority

perform **without arm support** (support may be

available for safety)

Walking (comfortable pace)

Walking (fast pace) for short duration (10 meters) Walking (fast pace) extended cardio (2 minutes)

Walking on toes

Walking on heels

Heel to toe (tandem) walking

Heel to toe (tandem) backwards walking

Walking backwards

Walking sideways- cross over

Walking sideways- side steps

Walking and changing directions (i.e., a turn of more than 45 degrees)

Walking with frequent starts and stops

Walking with head turns

Walking in different directions (i.e., a change of 45 degrees or less)

Walking and picking up objects

Walking while talking

Walking while holding a static object

Other, please specify:

27. In a typical class, which of the following **strength training exercises (i.e., using free weights and/or resistance bands and/or bodyweight only)** do the majority (>=50%) of your participants perform?

If yes, please check whether the majority (>=50%) of participants perform the exercise while standing or sitting.

Yes- and the majority perform

No while **standing**

Yes- and the majority perform while **sitting**

Legs (e.g., squats, lunges, etc.)

Core (e.g., plank, seated ab crunch, rows, etc.)

Chest (e.g., wall push-ups, chest press, etc.)

Arms (e.g., bicep curl, triceps extension, etc.)

Shoulders (e.g., overhead press, deltoid lateral raise, etc.)

Other, please specify:

28. Do you conduct any other exercises in the {{ contact.custom1 }} exercise program not mentioned in the

above lists?

29. Is there anything else you would like to tell us about the exercise content of the {{ contact.custom1 }}

exercise program?

Section 3: Exercise Content

30. Please describe the obstacle course exercise that you prescribe:

Section 4: Target population and inclusion/exclusion criteria

**The next section will focus on target population and inclusion/exclusion criteria of the exercise program. Please check the best answer and specify if needed.**

**If you teach multiple sessions/classes of the exercise program identified in the recruitment e-mail and throughout the survey, please think of all the sessions you teach of that program as a whole (i.e., not individual sessions/classes) when answering the questions.**

31. Does the {{ contact.custom1 }} exercise program target any specific older population? (check all that apply)

Healthy older adults

Older adults with a previous fall history

Older adults with a specific health condition (i.e. Parkinson’s, MS, arthritis, etc.) Other, please specify:

32. Are there any specific inclusion and/or exclusion criteria of the {{ contact.custom1 }} exercise program?

No

Yes

Section 4: Target population and inclusion/exclusion criteria

33. Please check all the inclusion and/or exclusion criteria that apply from the list below, or specify other criteria:

Minimum independence level (ex: walk independently, go to the washroom independently, etc.) Minimum strength level (ex: able to do the lowest modification of the exercise)

Completion of medical clearance (ex: valid PAR-Q, doctor’s note, etc.)

Minimum performance of specific tasks (ex: Standing on one leg for 2 seconds, standing for 20 minutes, etc.) Other, please specify:

34. Is there anything else you would like to tell us about the target population/inclusion and exclusion

criteria of the {{ contact.custom1 }} exercise program?

Section 5: Demographic Information

**The fifth and last section will focus on demographic information (i.e., location of the program and characteristics of the primary instructor of the program). The primary instructor is the individual who may be in charge of planning, coordinating and developing class content, teaching the majority of classes while monitoring individual progress and offering support and assistance, etc. Please check the best answer and specify if needed.**

**Please be advised that the information from the next two questions will not be used to identify programs.**

35. In which province/territory is the {{ contact.custom1 }} exercise program located?

36. What are the first three digits of the postal code of the location of the {{ contact.custom1 }} exercise program? **If you teach the {{ contact.custom1 }} exercise program at multiple locations, please provide the first three digits of the postal code of each location.**

If you need help finding the postal code of your program, please right click [here to](https://www.canadapost.ca/cpo/mc/personal/postalcode/fpc.jsf) open a postal code finder in a new tab.

Section 5: Demographic Information

**The next section will ask about your characteristics as the primary instructor of the exercise program. Please check the best answer and specify if needed.**

37. The {{ contact.custom1 }} exercise program is delivered by a:

Certified fitness instructor (i.e., CSEP, CAN-FIT-PRO, etc.)

Health professional (i.e., kinesiologist, physical therapist, etc.) Peer-leader

Other, please specify:

38. How many years of experience have you had instructing the {{ contact.custom1 }} exercise program?

1 or fewer

2 3 4 5

6 or more

39. What is your educational background/training? Check all that apply.

Exercise Physiology/Kinesiology degree

Physical therapy degree

Nursing degree

Fitness professional (i.e., Can-Fit Pro, CSEP) Other, please specify:

40. Have you received any specific training or education in falls prevention?

No

Yes

Section 5: Demographic Information

41. Please specify the name of the falls prevention course/ training program that you received:

42. Is there anything else you would like to tell us about your background?

End of Survey Question

43. Do you know of any other centres in your city/region that offer fall prevention/balance exercise programs for community dwelling older adults? If so, please provide the name of the centre below.

Permission to contact- Future Studies

**Please indicate by right clicking on the link below to open a new tab and providing your contact information that we may contact you to invite you to take part in future research on fall prevention and balance training community-based exercise programs for adults aged 50 years and older in Canada.**

**Clicking on the link below and providing your contact information does not imply that you consent to participate in subsequent research initiatives, it simply authorizes us to contact you.**

**Your name and contact information will be stored in a different database separate from your survey responses on a locked computer in a locked office in order to maintain the confidentiality of the questionnaire information you provide. After providing your contact information, please return to this page to complete the survey.**

**Please right click on the provided link to open a new tab if we may contact you to invite you to take part in future research: Permission to contact**

Summary of the Findings

**Please indicate by right clicking on the link below to open a new tab and providing your contact information if you would like to receive a summary of the findings of this study. Your name and contact information will be stored in a different database separate from your survey responses on a locked computer in a locked office in order to maintain the confidentiality of the questionnaire information you provide. After providing your contact information, please return to this page to complete the survey.**

**Please click on the provided link if you would like to receive a summary of the findings of this study: Summary of Findings**

End of Survey

**Based on your answers from the eligibility page, your exercise program is not eligible for this**

**study. Thank you for your participation and for the information you shared with us.**

**We would like to remind you that all information that you have provided us will be kept completely confidential.**

**If you have any questions, comments, or concerns we would be happy to speak with you. You may contact us at __________________ or __________________, or e-mailed at __________________ or __________________.**

**Thank you again for your time.**

End of Survey

**The survey is completed. Thank you for your participation and for the information you shared with us. What you shared will help us understand the fall prevention exercise programs available to older adults.**

**We would like to remind you that all information that you have provided us will be kept completely confidential.**

**If you have any questions, comments, or concerns we would be happy to speak with you. You may contact us at __________________ or __________________ , or e-mailed at __________________ or __________________.**

**Thank you again for your time.**
